# Effects of group mindfulness intervention on high-level distance runners: a quasi-experimental study

**DOI:** 10.3389/fspor.2025.1556404

**Published:** 2025-04-23

**Authors:** Bence Kelemen, Renátó Tóth, Ottó Benczenleitner, László Tóth

**Affiliations:** ^1^ School of Doctoral Studies, Hungarian University of Sports Science, Budapest, Hungary; ^2^ Institute of Sports Sciences, Eszterházy Károly Catholic University, Eger, Hungary; ^3^ Department of Psychology and Sport Psychology, Hungarian University of Sports Science, Budapest, Hungary; ^4^ Teacher Training Institute, Hungarian University of Sports Science, Budapest, Hungary

**Keywords:** mindfulness, distance running, sport psychology, performance, flow, competitive anxiety, emotion regulation

## Abstract

**Introduction:**

This study explored the impact of the Mindful Sport Performance Enhancement (MSPE) program, adapted for elite distance runners.

**Methods:**

Twenty Hungarian national and international-level athletes participated in a quasi-experimental design. The experimental group (*n* = 10) completed a six-week MSPE intervention, while the control group (*n* = 10) received no mental training. Psychological constructs, including flow, competitive anxiety, mindfulness, and emotion regulation, were assessed pre- and post-intervention.

**Results:**

Results showed significant improvements in the experimental group across most variables, particularly flow and cognitive anxiety, with no changes in the control group.

**Discussion:**

These findings highlight the effectiveness of a group-based mindfulness program in enhancing mental well-being and performance in elite runners. The scalable, structured format offers a practical alternative to traditional sports psychology approaches for high-performance athletes.

## Introduction

What are the most effective mental preparation protocols in long-distance running? The literature extensively describes well-established and proven training programs used in physical preparation, such as the Norwegian method, which is famous for its “double-threshold” training days, and the so-called “80:20”, or Polarized training distribution (1–4). In contrast, the area of sport psychology programs for elite runners has not been extensively researched. However, structured and systematic mental preparation in long-distance running is relevant from several perspectives. Similar to other competitive sports, maintaining motivation (5–7), ensuring mental well-being (which is increasingly critical for elite athletes today) (8, 9), and managing anxiety in high-stakes situations are essential for success (10–13).

Mindfulness, originally rooted in Buddhist meditation, was introduced into Western therapeutic frameworks through Jon Kabat-Zinn's Mindfulness-Based Stress Reduction (MBSR) program. This program aimed to reduce stress and enhance both physical and mental well-being (14). Over time, mindfulness has gained notable traction in elite sports, emerging alongside cognitive approaches such as Rational Emotive Behavior Therapy (REBT) (15, 16). Mindfulness is defined as a purposeful, non-judgmental awareness of the present moment, involving the observation of thoughts, emotions, and bodily sensations without attempting to change or suppress them (14). It encompasses two essential elements: self-regulated attention and openness to experience. Self-regulated attention involves the sustained focus on one's mental states, while openness to experience reflects a non-biased and accepting attitude toward internal and external experiences (17, 18).

The adoption of mindfulness in sports psychology became widely recognized through its practical application by NBA coach Phil Jackson. His success with teams such as the Chicago Bulls and Los Angeles Lakers subsequently inspired other teams, including the Seattle Seahawks, and individual athletes like Novak Djokovic (19). In contemporary sport psychology, two mindfulness-based intervention protocols have gained prominence: the Mindfulness-Acceptance-Commitment (MAC) approach and the Mindful Sport Performance Enhancement (MSPE) program. These approaches combine mindfulness with acceptance and commitment strategies to enhance athletic performance (20–22).

Traditional sports psychology, which emphasizes regulating psychological and physical states for optimal performance, has been critiqued for its reliance on techniques such as goal setting and imagery, which often yield inconsistent results in reducing anxiety and enhancing performance (20, 23). This inconsistency aligns with the “ironic process” theory, which suggests that efforts to suppress negative thoughts can paradoxically amplify them, disrupting focus and self-regulation (24, 25). In contrast, mindfulness-based approaches provide a promising alternative. By encouraging non-judgmental awareness, mindfulness allows athletes to detach from emotions and cognitions, thereby facilitating selective focus on task-relevant cues (18).

Mindfulness has been shown to counteract performance anxiety through several mechanisms. First, it enhances focus, enabling athletes to concentrate on task-relevant cues and minimize distractions during high-pressure situations (15, 26–28). Second, emotional regulation techniques, such as mindful breathing, help reduce physiological arousal and improve emotional stability (29, 30). Third, mindfulness fosters non-judgmental awareness, allowing athletes to accept intrusive thoughts without engaging with them, thereby preventing negative cognitive spirals (31–33). These mechanisms align with empirical evidence demonstrating that mindfulness optimizes focus and emotional resilience, key factors in athletic performance (34).

Mindfulness has demonstrated significant success in enhancing performance and psychological resilience, particularly among elite athletes (35, 36). Empirical studies have shown its effectiveness in improving emotional regulation, focus, and attention—key factors for success in high-stakes competitive environments. Mindfulness also facilitates flow, a state of complete immersion in an activity, which is associated with peak performance (37–40). Research across disciplines such as swimming, running, and archery further supports the positive relationship between mindfulness training and the achievement of flow (40–42). Additionally, mindfulness reduces anxiety and improves emotional regulation, promoting relaxation and aiding recovery between training sessions and competitions. These benefits not only optimize psychological readiness but also enhance physical regeneration, helping athletes maintain peak performance over time (43–45). The growing availability of online and mobile app-based mindfulness programs offers promising opportunities for scalable, accessible interventions tailored to elite athletes (46). These digital platforms facilitate consistent practice and integration into athletes' demanding schedules, making mindfulness a versatile and impactful tool for improving both performance and well-being in elite sports contexts (26, 34, 47–49).

In middle and long-distance running, athletes encounter various tactical challenges, including those in multi-round world championships requiring slower pacing or time-trial formats with evenly paced efforts (50–56). These scenarios require focus, adaptability, and emotional regulation (53, 57). Literature suggests that time trials benefit from a narrow focus (58, 59), while slow tactical races necessitate broader focus and quick decision-making in response to competitors' movements and pace changes (57, 60–62). Emotional regulation plays a significant role, as suggested by Noakes' Central Governor Model, which links internal and external reactions to pain perception and performance (63–66). This is particularly relevant in longer races, such as the marathon, where the “marathon wall” phenomenon occurs in the final third of the race (67–70). Several studies over the past two decades, including Corbally and Wilkinson's meta-analysis of 235 athletes, have shown promising results for long-distance runners; however, participants were recreational or collegiate runners (71). Long-distance running, with its unique psychological challenges like prolonged focus, fatigue management, and tactical decision-making, can benefit from mindfulness-based interventions that aid athletes in maintaining composure under fatigue and adapting pacing strategies in competitive environments.

Given the existing gap in research on elite distance runners, the present study sought to examine the effects of a group-based mindfulness-based sport psychology intervention on a sample of high-level long-distance runners (national and international levels). The MSPE program was selected for its targeted focus on optimizing flow states and incorporating task-specific mindfulness strategies directly relevant to the demands of athletic performance (34). Unlike broader interventions such as the Mindfulness-Acceptance-Commitment (MAC) approach, which offer more general mindfulness techniques, the MSPE program emphasizes practical applications that are specifically tailored to the psychological challenges faced by athletes, such as focus, emotional regulation, and performance under pressure. Its adaptability to group formats and elite sports contexts renders it particularly appropriate for high-level distance runners.

The hypothesis of this study posited that the MSPE intervention would result in significant improvements in mindfulness, flow, and emotional regulation, alongside reductions in cognitive and somatic anxiety when compared to a control group. In order to assess the effectiveness of the intervention, the study employed the FAME framework, which includes established psychological factors such as flow, competitive anxiety, mindfulness, and emotion regulation (22). Additionally, the intervention's outcomes were compared with those of a control group competing at a similar performance level to determine the relative effectiveness of the MSPE approach in fostering psychological and performance-related improvements.

## Methods

### Procedure

Participants were recruited by reaching out to Hungarian national-level long-distance runners, including those competing in national championships, as well as international athletes representing Hungary at the senior or junior national team level. Recruitment focused on individuals competing in distances between 800 meters and the marathon, with engagement facilitated through athletic clubs. For ethical reasons, a randomized control group was not feasible, so to assess the effectiveness of mindfulness, we employed a quasi-experimental design with a non-randomized control group. Participants in the control group did not undergo any organized sports psychology training during the research.

Both groups completed an online questionnaire package at the same intervals, which included general demographic information, and a set of validated Hungarian questionnaires designed to assess factors that, according to the literature, influence sports performance: flow, anxiety, mindfulness, and emotion regulation (FAME) (22). The pre-test assessment was conducted prior to the initiation of the intervention, while the post-test measurement was administered to both groups six weeks later, one week after the final mindfulness session. Potential biases associated with self-reported data, such as social desirability or recall bias, were mitigated by ensuring participant anonymity and standardizing the timing of questionnaire administration. Additionally, validated instruments with strong psychometric properties were used to enhance the reliability of the findings.

The mindfulness intervention was delivered using the Mindful Sport Performance Enhancement (MSPE) program, specifically adapted for runners. Sessions were held in an online group format in Hungarian, led by a trained MSPE instructor who also holds an MSc Degree in Athletics Coaching with a specialization in long-distance running. Participant recruitment took place between August 1, 2024, and September 20, 2024. The MSPE intervention was conducted from September 29, 2024, to November 3, 2024. Participants were informed about the details of the questionnaire administration and the sport psychology program. Written consent was obtained from all participants regarding both the handling of their responses and their participation in the program. The research was approved by the Ethics Committee of the Hungarian University of Sport Sciences. All subjects gave written informed consent in accordance with the Declaration of Helsinki.

### Participants

The study sample consisted of 20 athletes (*N* = 20). We determined the required sample size using G*Power 3.1.9.7 software. The power analysis parameters [effect size f = 0.40, α error probability = 0.05, power (1-β) = 0.80, *p* = 0.5] were set based on the large effect sizes reported for anxiety reduction in mindfulness programs, as reviewed by Myall and colleagues (72). According to these calculations, a minimum total sample size of 16 participants was required to achieve adequate statistical power. The mindfulness group included 10 participants (6 males and 4 females), with an equal distribution of athletes competing at both national (*n* = 5) and international (*n* = 5) levels. The average age in this group was *M* = 25.00 years (*SD* = 2.00 years), and they had a mean running experience of *M* = 12.3 years (*SD* = 5.21 years). Within the mindfulness group, three athletes reported engaging in various forms of mental training as part of their preparation, although none had previous experience specifically with meditation or mindfulness practices.

Similarly, the control group comprised 10 athletes (7 males and 3 females), also evenly divided between national-level (*n* = 5) and international-level (*n* = 5) competitors. The mean age of participants in this group was *M* = 25.4 years (*SD* = 6.10 years), and their average running experience was *M* = 11.15 years (*SD* = 6.20 years). Two athletes in the control group also participated in mental training during their training process; however, none had prior exposure to meditation or mindfulness techniques.

### Mindfulness intervention

In this study, we implemented the Mindful Sport Performance Enhancement (MSPE) program, developed by Kaufman, Glass, and Arnkoff (34), in an online, group-based format. The MSPE program is grounded in Kabat-Zinn's (99) Mindfulness-Based Stress Reduction (MBSR) and Segal et al.'s Mindfulness-Based Cognitive Therapy (MBCT) (14, 73). Its primary objectives are to enhance athletes' flow experiences, boost self-confidence, improve attention focus, and regulate emotions while simultaneously reducing performance-related anxiety. The intervention was conducted over six weeks, comprising weekly 90 min sessions that incorporated a range of techniques, including diaphragmatic breathing, mindful eating, breath-focused sitting meditation, progressive body scanning, mindful yoga, and sport-specific running meditation (see Table 1).

**Table 1:** Overview of the MSPE protocol.

Session	Key ideas	Experiential progression	Conceptual progression
Session 1	Defining mindfulness; Nonjudgment; Rationale for MSPE; Getting off of automatic pilot	Candy Exercise; Diaphragmatic Breathing; Sitting Meditation with a Focus on the Breath	Why mindfulness is relevant for sport performance (e.g., flow); Foundational sedentary practice
Session 2	Overcoming practice obstacles; Core Performance Facilitators; Present-moment attention	Body Scan; Sitting Meditation with a Focus on the Breath review	How MSPE impacts sport performance; Mindfully moving attention
Session 3	Expectations; The body as a route to awareness	Mindful Yoga; Sitting Meditation with a Focus on the Body as a Whole	What gets in the way of mindful attention; Mindfulness of novel physical movement
Session 4	Attachments; Letting go; Acceptance versus resignation	Mindful Yoga review; Walking Meditation	What powers judgments and expectations; Mindfulness of familiar physical movement
Session 5	Non-striving and bare awareness; Choice in self-care	Sport Meditation; Sitting Meditation with a Focus on the Breath, Body, and Sound	How to respond mindfully to expectations/attachments; Mindfulness of integral movements in sport(s) of focus
Session 6	Reflections on MSPE; Integrating a long-term mindfulness training routine	Body Scan review; Sport Meditation review; Gratitude discussion	Embracing a holistic perspective on training; Making dual commitments to physical and mental training

Participants engaged in both formal and informal mindfulness practices, utilizing exercises based on the 3R acronym (recognize, release, and reconnect) (74) as part of their daily homework assignments. Adherence to these practices was monitored through participants' journals, where they documented their experiences. At the beginning of each session, the instructor facilitated a discussion of the weekly homework assignments and reviewed the participants' weekly journals to track progress. Weekly feedback and reminders were provided to reinforce engagement, promoting the integration of mindfulness techniques into participants’ daily routines. At the conclusion of the program, participants completed a satisfaction survey to assess their adherence. The final session focused on the incorporation of mindfulness practices into daily life, with an emphasis on journaling, reminders, and cues to be utilized during training.

Building on the framework presented in Kaufman et al.'s Mindful Sport Performance Enhancement (MSPE) manual, the intervention was specifically adapted for runners (22). This adaptation included incorporating sport-specific practices that utilized running-related anchors during mindfulness exercises performed while running. Specifically tailored for high-level distance runners, the program addressed situational focus strategies relevant to training contexts, such as recovery and interval sessions, as well as tactical considerations for competitive scenarios, including pack running in championship-style races and drafting during personal best attempts in time trials. Additionally, significant attention was devoted to managing pre-race anxiety, processing post-competition results, and practicing relaxation techniques throughout the program.

### Measures

#### Flow state questionarre

The ability to experience flow was assessed using the Hungarian “Flow State Questionnaire (FSQ)” which has been validated in Hungarian (abbreviated as “FÁK”). The development of this questionnaire was based on Mihály Csíkszentmihályi's phenomenological definition of flow (39). The FSQ comprises 20 items and employs a two-factor solution: (1) “challenge-skill balance” (11 items) and (2) “task absorption” (9 items). Participants responded on a 5-point Likert scale, where 1 = “Does not describe me at all” and 5 = “Describes me completely”. The identification of these two factors supports the theoretical assumption that the fundamental determinants of flow are the balance between challenge and skill and immersion in the task. The Hungarian Flow State Questionnaire (FSQ) demonstrated good psychometric properties, including strong internal consistency (Cronbach's alpha = 0.89), a two-factor structure (challenge-skill balance and task absorption), and significant correlations with related constructs, establishing it as a reliable and valid tool for assessing flow experiences (75).

#### Competitive anxiety scale-2 (CSAI-2 h)

Competive anxiety was assessed using the Competitive State Anxiety Inventory-2 [CSAI-2; (76)], with a Hungarian validation developed by Sipos et al. (100), later revised by Tóth and collegues (in press). This assessment tool comprises an 18-item modified version that is categorized into three distinct scales: cognitive anxiety, somatic anxiety, and self-confidence. We excluded self-confidence from the analysis as it was not relevant to the research. Participants provide ratings on a 4-point Likert scale, where 1 denotes “not at all” and 4 signifies “very much”. The Hungarian adaptation demonstrated robust internal consistency, with Cronbach's alpha coefficients ranging from 0.7 to 0.8, indicating reliable measurement properties across the scales (77).

#### Mindful attention awarness scale (MAAS-H)

To assess mindfulness, we administered the Hungarian version of the Mindful Attention Awareness Scale (MAAS), originally developed by Brown and Ryan, a widely utilized tool for evaluating dispositional mindfulness (78). The scale consists of 15 items designed to capture an individual's capacity to focus on the present moment in a receptive and non-judgmental way. The items include both general and situationally specific prompts to gauge the frequency of mindful awareness. Each item is reverse-scored on a 6-point Likert scale (ranging from “almost always” to “almost never”), asking respondents to indicate how often they find themselves acting automatically or without attention. The Hungarian version of the Mindful Attention Awareness Scale (MAAS-H) demonstrated good internal consistency (Cronbach's alpha = 0.78), test-retest reliability (0.71 over two months), a single-factor structure, and valid correlations with affectivity and well-being measures, aligning with the psychometric qualities of the original scale (79).

#### State difficulties in emotion regulation scale (S-DERS)

The State of Emotion Regulation Difficulties Scale (S-DERS) is a 21-item self-report instrument designed to assess difficulties in emotion regulation. Respondents indicate their level of agreement on a 5-point Likert scale, where 1 corresponds to “Not at all” and 5 signifies “Completely”. The items within the S-DERS were adapted from the Difficulties in Emotion Regulation Scale (DERS) (80), which focuses on specific challenges individuals face in regulating their emotions. In contrast to the original DERS, which measures trait-based emotion regulation, the S-DERS specifically assesses state-based difficulties in emotion regulation. This distinction allows the S-DERS to account for short-term influences, including interpersonal experiences, situational variables, cognitive processes, and other emotional factors that may affect an individual's ability to regulate their emotions. The total scale of the State Difficulties in Emotion Regulation Scale (S-DERS) demonstrated excellent internal consistency with a Cronbach's alpha of 0.95, indicating high reliability across the full measure (81). The questionnaire was adapted into Hungarian for the research according to the recommendations in the literature (82).

#### Satisfaction scale

The experimental group, in addition to completing the aforementioned questionnaires, was also asked to fill out a brief satisfaction survey upon the conclusion of the intervention. This survey included the following inquiries: “To what extent did you find the MSPE course beneficial, and to what degree were you able to integrate the techniques learned into your practice?” and “How satisfied were you with your personal engagement in weekly/home-based exercises?” Respondents were asked to rate their responses on a 5-point Likert scale (1 = Not at all, 5 = Completely). Additionally, two open-ended questions were included: “If applicable, please describe a situation or occasion in which you successfully applied a technique learned in the course during athletic activities (training or competition)”; and “If applicable, please describe a situation or occasion in which you successfully applied a technique learned in the course in your daily life”.

## Scoring procedures and variable justification

Based on a general guideline suggesting that the sample size should be at least 5–10 times the number of variables studied (83), and considering the research hypotheses, the study included several variables, which necessitated primarily calculating total scores, except for the CSAI-2HR, where distinguishing between cognitive and somatic anxiety was relevant. For the FSQ, MAAS, and S-DERS questionnaires, total scores were calculated by summing the item scores, with the MAAS including reverse-scored items, which were adjusted following the prescribed scoring guidelines. In the case of the CSAI-2HR, the self-confidence subscale was excluded as it was not relevant to our research question, and the mean scores of the cognitive and somatic anxiety subscales were used for analysis. No missing data were present for any of the questionnaires.

### Statistical data analyses

Normality was assessed for each measurement, and all variables followed a normal distribution (skewness = −2–2; kurtosis = −7–7). Descriptive statistics were subsequently calculated. To explore the reliability, an internal consistency test was performed, with Cronbach's alpha coefficient greater than 0.60 considered acceptable (84). In our main analyses, we conducted repeated measures ANOVA, where the between-subject factors were the two groups (experimental and control), and the within-subject variable (time) was represented by the results of the five aforementioned variables in the pre- and post-test. This analysis is suitable for examining the main effects, such as the differences in outcomes between the two groups over time, the interaction effect (time*group), and the differences between the groups. All statistical analyses were performed using SPSS version 27.0.

## Results

### Descriptive statistics and Pre-intervention comparisons

Means and standard deviations are presented in Table 2. The final dataset contained no missing values or extreme outliers, and the assumptions of homogeneity and normality were satisfied. The descriptive statistics indicate no significant differences between the two groups in terms of age, years of running experience, performance level, race distance, or gender. Independent samples *t*-tests confirmed no significant differences between the groups at pre-test on any of the psychological indicators assessed (*p* > 0.05).

**Table 2:** Desciptive statistics.

Variable	MSPE (*n =* 10)				Control (*n* = 10)			
	Pre		Post		Pre		Post	
	M	SD	M	SD	M	SD	M	SD
Age	25.00	2.11	25.00	2.11	25.40	6.43	25.40	6.43
Experience	12.30	5.50	12.30	5.50	11.15	6.64	11.15	6.64
FSQTOT	76.10	7.82	87.20	5.27	77.70	7.65	74.40	12.19
CA	2.17	0.94	1.42	0.36	2.20	1.00	2.18	1.04
SA	2.32	0.74	1.68	0.59	2.07	0.65	1.97	0.48
MAASTOT	54.40	13.30	59.90	9.05	55.00	9.32	53.10	10.07
SDERSTOT	54.70	11.48	48.70	4.57	50.20	8.04	48.10	8.88

FSQTOT, flow state questionnaire total score; CA, cognitive anxiety; SA, somatic anxiety; MAASTOT, mindful attention awereness scale total score; SDERSTOT, state difficulties in emotion regulation scale total score.

The internal consistency of the questionnaires was evaluated at both pre-test and post-test using Cronbach's alpha. At pre-test, the internal consistency values were: flow-state (Cronbach's α = 0.76), cognitive anxiety (Cronbach's α = 0.93), somatic anxiety (Cronbach's α = 0.84), mindfulness (Cronbach's α = 0.77), and emotion regulation (Cronbach's α = 0.76). At post-test, the internal consistency values were: flow-state (Cronbach's α = 0.91), cognitive anxiety (Cronbach's α = 0.94), somatic anxiety (Cronbach's α = 0.71), mindfulness (Cronbach's α = 0.73), and emotion regulation (Cronbach's α = 0.67). Across both time points, the internal consistency of the scales was acceptable (Cronbach's α ≥ 0.60) to good (Cronbach's α ≥ 0.70), indicating reliability of the constructs measured (85).

## Result of MSPE intervention

### Flow-state

The main effect of time was not statistically significant [F(1,18) = 3.65, *p* = 0.072]. This suggests that, overall, there was no substantial difference between pre- and post-test scores across groups. However, the partial eta squared (*η*^2^ = 0.17) indicates a medium effect size, suggesting a trend toward change that might become significant with a larger sample size. In contrast, the interaction effect between time and group was statistically significant, F(1,18) = 12.45, *p* = 0.002). This indicates that the change in scores over time differed significantly between the experimental and control groups. The large effect size (*η*^2^ = 0.41) highlights the strong dependence of time-related changes on group membership.

The between-subjects main effect of group was not statistically significant F(1,18) = 2.947, *p* = 0.10). This suggests that the average scores did not differ significantly between the experimental and control groups. However, the medium effect size (*η*^2^ = 0.14) indicates a potential trend toward group differences that may require further exploration or a larger sample size for confirmation.

### Cognitive anxiety

The main effect of time was statistically significant [F(1,18) = 11.51, *p* = 0.003]. This indicates a significant difference between pre- and post-test scores across groups, with a large effect size (*η*^2^ = 0.39), suggesting that the intervention had a substantial impact over time. The interaction effect between time and group was also statistically significant [F(1,18) = 10.534, *p* = 0.004]. This finding highlight that the change in scores over time differed significantly between the experimental and control groups. The large effect size (*η*^2^ = 0.37) emphasizes the strong influence of group membership on the observed time-related changes.

The main effect of group was not statistically significant [F(1,18) = 1.13, *p* = 0.30]. This suggests that the average scores did not differ significantly between the experimental and control groups. The small effect size (*η*^2^ = 0.06) further supports this conclusion.

### Somatic anxiety

The main effect of time was statistically significant [F(1,18) = 6.97, *p* = 0.017]. This result indicates a significant difference between pre- and post-test scores, with a large effect size (*η*^2^ = 0.28), suggesting notable changes over time in the measured outcomes. The interaction effect between time and group was not statistically significant [F(1,18) = 3.69, *p* = 0.07]. Although the *p*-value approached significance, the effect size (*η*^2^ = 0.17) suggests a medium effect, indicating a potential trend in how changes over time might differ between the experimental and control groups. This warrants further investigation with a larger sample size to determine whether this trend represents a meaningful effect.

The between-subjects main effect of group was not statistically significant [F(1,18) = 0.005, *p* = 0.95]. This result confirms that the average scores did not differ significantly between the experimental and control groups. The near-zero effect size (*η*^2^ = 0) supports the absence of meaningful group-level differences in the overall scores.

### Mindfulness

The main effect of time was not statistically significant [F(1,18) = 1.47, *p* = 0.241]. This suggests that there was no significant change between pre- and post-test scores across groups, and the small effect size (*η*^2^ = 0.08) indicates limited time-related variance in the measured outcomes. However, the interaction effect between time and group was statistically significant [F(1,18) = 6.212, *p* = 0.02]. This finding indicates that changes over time differed significantly between the experimental and control groups. The medium-to-large effect size (*η*^2^ = 0.26) suggests that group membership played a notable role in shaping the observed time-related changes.

The main effect of group was not statistically significant [F(1,18) = 0.48, *p* = 0.50]. This indicates that the average scores did not differ significantly between the experimental and control groups. The very small effect size (*η*^2^ = 0.03) supports the absence of substantial group-level differences in overall scores.

### Emotion regulation

A significant main effect of time was observed F(1,18) = 6.780, *p* = 0.02). This finding indicates that there were significant changes in the measured outcomes between the pre- and post-test conditions. The moderate effect size (*η*^2^ = 0.27) suggests that time had a meaningful impact on the scores. However, the interaction effect between time and group was not statistically significant, [F(1,18) = 1.572, *p* = 0.23]. This indicates that the changes over time did not differ significantly between the experimental and control groups. The small effect size (*η*^2^ = 0.08) supports the absence of a strong interaction effect.

**Figure 1:**
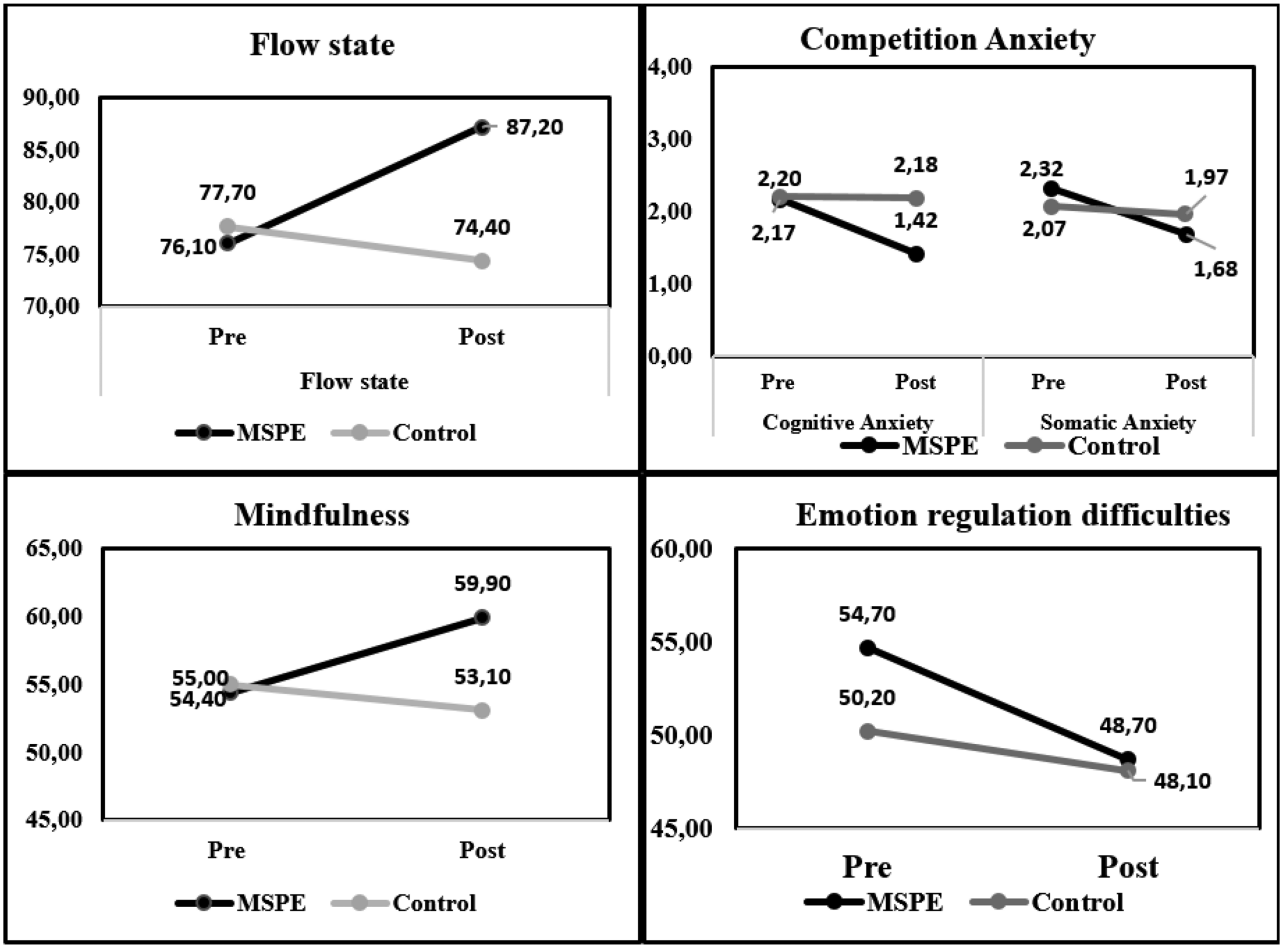
Changes in measured psychological variables.

The main effect of group was not statistically significant [F(1,18) = 0.53, *p* = 0.48]. This suggests that there were no significant differences in the overall average scores between the experimental and control groups. The small effect size (*η*^2^ = 0.028) confirms that group membership did not strongly influence the outcomes. The temporal changes in the measured psychological variables are presented in Figure 1.

### Feedback on the intervention

After the conclusion of the intervention, in response to the question regarding the perceived usefulness of the intervention, the participants provided a rating of M = 4.3 (SD = 0.45) on a 5-point Likert scale. Regarding their satisfaction with their own weekly formal and informal practice, they rated it M = 3.9 (SD = 0.7) on the same 5-point scale. The following longer, more detailed responses from participants reflect their experiences during training and competition: “The 3R technique has been very effective since the course began. For example, the wind used to frustrate me during training, but now I handle it much better. When factors like fatigue or weather affect my training, I can adjust more easily, and it no longer causes problems”. Another participant noted, “Breathing technique: focusing while running. Letting go: accepting and releasing negative feelings. This includes not clinging to expectations, like insisting things go exactly as planned”. One runner shared, “During training, when the wind was strong and I was tired, negative thoughts emerged after the second run. I was able to apply what we practiced to refocus, finish the session, and I felt proud afterward”. Another participant stated, “I find it easier to concentrate on the task at hand”. Another mentioned, “During both competitions and training, I focus on my arm movements to redirect my attention from negative thoughts. In races, I focus on the runner ahead of me”. Finally, one participant remarked, “During treadmill sessions, I switched my focus between different body parts, and the sessions passed much more quickly”.

For example, when participants were asked about the application of the techniques in their daily routines, they offered the following responses: “The meditation and diaphragmatic breathing exercises have been effective in managing stressful situations at work”. Another participant shared, “In everyday life, I often remembered the diaphragmatic breathing practice during emotionally tense situations. Whenever the situation allowed, I tried to perform the exercise as soon as possible, which greatly helped me cope with stress”.

## Discussion

Prior to the intervention, no significant differences were found between the experimental and control groups regarding demographic variables (age, gender) or athletic characteristics (competitive level, race discipline, sporting age). Moreover, there were no significant differences in any measured psychological variables. These findings confirm the validity of the experimental design and the legitimacy of the results obtained. The study revealed several key findings regarding the intervention's impact on flow, anxiety, mindfulness, and emotion regulation. Changes in flow and mindfulness were strongly influenced by group membership, with the experimental group showing more significant improvements over time compared to the control group. Cognitive and somatic anxiety demonstrated notable reductions across the intervention period, particularly in the experimental group, highlighting the program's effectiveness in managing both types of anxiety. Emotion regulation also improved over time, though these changes were not specific to group membership. Overall, the results underscore the intervention's potential to enhance psychological states crucial for performance and well-being.

The results of our study on the Mindful Sport Performance Enhancement (MSPE) program are consistent with previous research in mindfulness and sport psychology, including studies involving less experienced runners. Specifically, we observed significant improvements in several key psychological factors (FAME framework), including increased mindfulness, reduced anxiety, and enhanced self-confidence, all of which are consistent with findings from other studies examining mindfulness-based interventions in sports (31, 41, 86). Our results also revealed a notable increase in athletes' ability to enter flow states, a critical factor for facilitating peak performance (34, 37). These improvements suggest that mindfulness practice can contribute to optimal performance by enhancing an athlete's capacity to focus, maintain calm under pressure, and remain fully engaged in the present moment (40, 87). Furthermore, our study found that mindfulness training improved emotional regulation, particularly by reducing the frequency of negative thoughts and emotions (significantly lower scores in the non-acceptance and clarity subscales) and enhancing the ability to recognize those negative patterns (increased awareness) to manage them effectively. This is consistent with the notion that mindfulness promotes non-judgmental awareness and acceptance of one's thoughts and emotions, which helps to reduce rumination and anxiety (26, 86, 88, 89). In line with the work of Bülmayer et al. (31), who reported that mindfulness interventions help athletes to manage distressing thoughts, our results emphasize the importance of emotional regulation for maintaining mental resilience in competitive sports. The ability to manage negative thoughts and emotions can further support performance by reducing the cognitive and emotional interference that can undermine focus and motivation, particularly under stressful or high-pressure situations (72, 90). Moreover, by enhancing self-regulation, mindfulness may help athletes to avoid the negative spirals of anxiety and self-doubt that often occur in performance contexts (30).

As part of the program, participants learned to identify and let go of difficult emotions and intrusive thoughts, not only in their sports context but also in their daily lives. This skill helped them step out of “autopilot mode” and avoid becoming trapped in negative thought spirals (91). This is particularly important given the increasing attention to the chronic performance pressure and stress faced by elite athletes, which often leads to burnout (92–95). The mindfulness-based approach fosters a more balanced and harmonious lifestyle, which can indirectly enhance both athletic performance and enjoyment. By helping athletes manage stress and disengage from counterproductive mental patterns, mindfulness provides a pathway to greater well-being and sustained peak performance (96).

In our previous research with Hungarian national long-distance runners, we observed that mental preparation did not have a significant modulatory role in the anxiety categories measured by the CSAI-2 (97). This could be attributed to the small sample size and the inconsistent application of sport psychology preparation methods. It is also possible that the role of ironic processes, as described by Wegner (25), played a part, since none of the athletes utilized mindfulness or cognitive-based techniques in their preparation. Additionally, the literature suggests that high-level athletes with extensive careers may have developed coping strategies during their training process that produce similar effects, even without direct interventions. In our subsequent study, conducted with a larger international sample, we found that neither sports participation nor specific mental preparation techniques had a significant impact on performance outcomes or anxiety levels. In our later research, conducted with a larger international sample, we found that neither gender, level of sports participation (recreational vs. national vs. international), nor race discipline had an impact on performance-enhancing mental skills. However, mental preparation itself was found to have a significant effect (98). The current study supports this observation.

While the psychological benefits of the MSPE program were thoroughly assessed, the relationship between these benefits and actual performance metrics, such as race times or competitive outcomes, requires further investigation. Although direct performance measures were not explicitly evaluated in this study, the psychological changes observed—such as enhanced focus, reduced anxiety, and improved emotion regulation—are well-documented as factors that positively influence athletic performance, including better tactical decision-making, sustained effort, and resilience. Throughout the training, participants regularly discussed and reflected on their performance outcomes during online sessions, highlighting how the psychological gains influenced their approach to competition. While external factors, such as pace, weather conditions, and momentary form, may obscure the direct impact on race results, many participants indicated in the satisfaction survey that they felt better able to reach their potential. This perception of enhanced self-efficacy was likely also acknowledged by coaches and teammates, who observed increased effort and contributions. Future research should incorporate objective performance metrics to further substantiate the links between psychological development and performance outcomes in competitive settings.

The findings of this research have important practical implications for enhancing athletic performance. The MSPE protocol demonstrated its effectiveness in improving athletes' mental preparation and performance across various stages. Prior to competitions, the program aids in anxiety management, while during competition, its focus on present-moment awareness enables athletes to shift focus, regulate emotions, and make tactical decisions in real time. Additionally, the protocol supports recovery, relaxation, and the processing of outcomes, whether success or failure. With its structured, six-week time frame and adaptability to group formats, including online delivery, the program offers a more accessible alternative to traditional one-on-one sports psychology interventions, which often require indefinite time commitments. However, its success relies on athletes' commitment to consistent practice and the independent development of mental skills. This research highlights the potential for MSPE to become a scalable and structured tool that can be integrated into training regimens to help athletes manage pre-race anxiety, maintain focus during competition, and recover effectively post-race. Its online format further extends its reach, making it accessible to athletes with demanding schedules or limited access to in-person services. Moving forward, future studies should explore the long-term effects of the MSPE program, including how athletes integrate mindfulness practices into their daily lives, to better understand the sustainability and broader impact of the program on athletic performance and psychological well-being.

## Strenght and limitations

A key strength of this study lies in its novel application of the intervention among high-level athletes (national and international level), making it, to our knowledge, the first of its kind. Moreover, high-level athletes were also included as a control group, which further strengthens the comparative rigor. Previous mindfulness-based interventions for runners have predominantly targeted lower-level or recreational athletes. The intervention's demonstrated impact across multiple psychological domains highlights its effectiveness and relevance for this population.

Despite its contributions, this study has several notable limitations. The small sample size (*N* = 20) and non-random allocation of participants to the control group limit the generalizability of the findings. The small sample was largely due to the specific inclusion criteria, which restricted the pool of eligible high-level athletes in Hungary, and the reluctance of some athletes to participate. Additionally, ethical considerations prevented the use of random assignment, as it would have been unethical to deny participation to volunteers or reassign them to a control group. The reliance on self-reported data from questionnaires is another limitation, as it may introduce response biases. While anonymity was assured to encourage honest responses, the self-reported nature of the data still poses potential limitations in terms of accuracy. Future studies should aim for larger, randomized samples to enhance external validity, and the inclusion of active control groups would help establish stronger causal links between the intervention and observed outcomes. Longitudinal follow-up studies are also recommended to assess the sustainability of the intervention's effects over time. To ensure the reliability of the results, future research should control for potential confounding variables, such as training volume, recovery practices, and external stressors during the intervention period, to better isolate the effects of the mindfulness intervention. Additionally, future research could benefit from examining the effects of mindfulness through mediation analyses, which would allow for a more detailed exploration of its anxiety-reducing properties or its role in enhancing flow states.

## Conclusion

This study implemented a mindfulness-based group sports psychology intervention among high-level, national and international distance runners with extensive competitive experience. In conclusion, the intervention proved effective in enhancing key psychological states, particularly flow, mindfulness, and anxiety management, with the experimental group showing more pronounced improvements compared to the control group. While emotion regulation also improved over time, these changes were not group-specific. These findings emphasize the potential of targeted interventions to foster mental skills critical for performance and well-being in this population. Limitations of this study include the non-random nature of the control group and a relatively small sample size. Nonetheless, the positive outcomes strongly encourage further research involving larger samples, active and randomized control groups, and longer-term follow-up. Additionally, practical applications of the MSPE intervention tailored specifically for distance runners are warranted for enhancing performance, anxiety management, and mental well-being. This approach, with its group format, structured duration, and present-moment focus, is not only effective but also more accessible to a broader range of athletes compared to traditional one-on-one sports psychology methods.

## Data Availability

The original contributions presented in the study are included in the article/Supplementary Material, further inquiries can be directed to the corresponding author.
